# Mediation and Moderation Effect of Psychosocial Factors on the Relationship Between Health Literacy and Well-Being in Adolescents

**DOI:** 10.3390/pediatric18010029

**Published:** 2026-02-12

**Authors:** Tania Gaspar, Marina Carvalho, Miguel Arriaga, Barbara Sousa, Margarida Gaspar-Matos

**Affiliations:** 1 Digital Human-Environment Interaction Labs (HEI-LAB), Lusófona University, Campo Grande, 1749-024 Lisbon, Portugal; barbara.f_vils@hotmail.com; 2 CHRC, Lisbon NOVA University, 1169-056 Lisbon, Portugal; 3 Faculty of Medicine, Aventura Social, Institute of Environment Health (ISAMB), University of Lisbon, 1649-004 Lisbon, Portugal; marina.carvalho@ismat.pt (M.C.);; 4 Instituto Superior Manuel Teixeira Gomes, 8500-656 Portimão, Portugal; 5 Direção-Geral da Saúde, 1049-005 Lisbon, Portugal; miguelarriaga@dgs.min-saude.pt; 6 Aventura Social-Associação, SPIC, 1675-184 Lisbon, Portugal; 7 Faculdade de Ciências Humanas, Catholic University, 1649-023 Lisbon, Portugal

**Keywords:** youth, public health, socio-economic, family, school, mental health

## Abstract

Purpose: Higher health literacy is associated with better health behaviors and better overall well-being; however, the contribution of relational and socio-economic factors to this association remains insufficiently explored. The present study aimed to examine the relationships between health literacy, well-being, social support, and stress among adolescents. In particular, the mediating roles of social support (family, peers, and teachers) and stress in the association between health literacy and well-being were analyzed. Participants and Methods: Data were drawn from the 2022 wave of the Health Behaviour in School-aged Children (HBSC) study, an international survey conducted every four years in collaboration with the World Health Organization (WHO) and implemented according to a standardized protocol. The sample comprised 7643 students from the 6th, 8th, 10th, and 12th grades of Portuguese public schools. Of the participants, 53.9% were female, and the mean age was 15.05 years (SD = 2.36). Gender-based comparisons indicated statistically significant differences for all study variables, with the exception of health literacy. Results: Mediation analysis reveals an effect of health literacy on well-being. After the inclusion of the mediating variables, the direct effect of health literacy on lack of well-being remained negative. All four mediators showed statistically significant indirect effects, accounting for the difference between the total and direct effects. These findings indicate that the association between health literacy and lack of well-being was partially mediated by family support, peer support, relationships with teachers, and stress. Health literacy influenced lack of well-being both directly and indirectly through these mediating pathways, with stress emerging as the strongest indirect contributor. Conclusions: The findings support an ecological interpretation of health literacy and well-being, as these constructs are embedded within multiple interacting systems. Individual adolescent characteristics, such as gender, age, and stress management, are interconnected with interpersonal contexts, including relationships with family members, peers, and teachers. In addition, adolescents’ socio-economic circumstances appear to play a relevant role in shaping both health literacy and perceptions of well-being.

## 1. Introduction

Health literacy (HL), defined as the ability to find, understand and effectively use information to support health-related decisions and actions [1], is a key factor for well-informed choices throughout an individual’s lifespan. In particular, childhood and adolescence are both stages of human development in which children and adolescents, due to biological, social and individual factors, are more prone, particularly in stressful situations, to perform risk behaviors [2], which are, in turn, founded in decision-making processes [3]. Therefore, HL in childhood and adolescence plays a particularly relevant role in these age groups. However, studies conducted with children and adolescents show low to moderate [4,5] levels of HL in this population.

Health outcomes are shown to be related to HL. A meta-analysis conducted by Fleary, Joseph and Pappagianopoulos [6] suggested the existence of an important association between HL and health behaviors in adolescents. Also, other studies provided evidence of a positive relationship between HL and well-being in adolescents [7,8]. However, a systematic review and meta-analysis did not find an association between HL and well-being in this population [9], suggesting that the mechanisms underlying this relationship in adolescents are not consistently understood. More recently, a meta ethnography carried out by Seidl and colleagues [10] showed thar HL was associated with psychological (e.g., stress, self-efficacy, self-control), contextual (e.g., social support) and sociodemographic factors (e.g., gender and age).

Social determinants of health influence the levels of HL [11,12], and the literature on this subject emphasizes the relevance of demographic, individual and contextual factors, among others, which interact to explain the complexity of child and adolescent HL [12]. Although there are several models [13], HL can be conceptualized according to a micro, meso and a macro level (based on the socioecological theory [14] and within the social–ecological framework of adolescent HL [10,15]).

At a meso (contextual) level, relationships with significant others are of most relevance in adolescence [16]. Parental and other family relationships, as well as relationships with peers and adults at school, are regarded as the main socialization sources during childhood and adolescence. HL levels and health behaviors can be promoted or hindered through attitudes, social norms and social support [11], and parents and peers can assume a modeling role on health or risk behaviors [17,18,19]. Also, at a micro (individual) level, HL is negatively related to stress [20], and stress management skills are positively related to well-being [21].

Studies demonstrate that individual differences in well-being can be explained by demographic factors. Gender and age have been shown to influence adolescents’ well-being [7,8,22,23,24]. A different pattern of results was obtained when explaining individual differences in HL. In this case, in one of the Health Behavior in School-aged Children (HBSC) studies, carried out in 10 European countries, gender was not associated with health literacy in most of the countries, whereas social economic status (SES) was positively related to HL [23].

Adolescence is full of challenges and stress factors. In order to promote greater well-being during this stage of growth, we have observed a correlation between greater well-being, high health literacy, and a greater perception of stress management skills [3,10,20,21].

Despite this, studies on the relationship between HL and well-being in adolescence are still scarce. Furthermore, the literature suggests that the existence of different mediator variables and individual differences can be found when studying the association between HL and well-being.

Based on this, the present research was aimed at analyzing the relationships between HL, well-being, social support and stress in adolescents. Specifically, we intended to study the mediator role of social support (family, peer and teachers) and stress in the relationship between HL and well-being. It is hypothesized that social support from family, friends and teachers, as well as stress management skills, will mean that health literacy can have an even stronger relationship with adolescents’ well-being.

## 2. Materials and Methods

### 2.1. Participants

The sample included 7643 students from the 6th, 8th, 10th, and 12th school grades, of which 53.9% were female, with an average age of 15.05 (*SD* = 2.36). The sample was representative of the school grades under study.

### 2.2. Instrument and Procedures

This study draws on data from the 2022 wave of the Health Behaviour in School-aged Children (HBSC) survey, an international study conducted every four years in collaboration with the World Health Organization (WHO) and implemented according to a standardized international protocol [25,26,27]. In Portugal, the HBSC survey has been coordinated and implemented since 1998, following the mandatory procedures for translation, cultural adaptation, and validation of the instruments (Table 1).

The sample is nationally representative and was obtained through a random, stratified sampling design based on the five main geographical regions of the country. Schools and classes were randomly selected within each stratum. Eligible participants were students attending the 6th, 8th, 10th, and 12th grades in Portuguese public schools. Participation required written informed consent from parents or legal guardians, and student participation was entirely voluntary. The overall response rate for the survey was 76.43%. The study includes students from 6th grade until 12th grade; some were over 18 years old, but we decided to include them and maintain the criterion of school grade.

In addition to the core HBSC indicators, several complementary variables included in the HBSC/WHO protocol were analyzed, namely health literacy, stress management, relationships with friends, difficulties in relationships with teachers, family support, and subjective well-being. Detailed descriptions of these complementary measures are available elsewhere [25,26].

### 2.3. Ethical Approval and Data Collection Procedures

The HBSC/WHO study aims to examine adolescents’ health behaviors and lifestyle patterns within their social contexts, as well as their associations with health and well-being outcomes. In Portugal, the 2022 HBSC survey received ethical approval from the relevant Ethics Committee (reference no. 281/21) and authorization from the General Directorate of Education and Science Statistics by 25 November 2021. Data collection was conducted in school settings, in accordance with the HBSC protocol. The questionnaire was administered online and completed anonymously by the students.

### 2.4. Statistical Analysis

Data analysis and processing were performed using SPSS 30.0 and JASP 0.18.1 software. Descriptive statistics were calculated for the variables under study, followed by a comparison of groups using Student’s *t*-test. Next, a Pearson correlation analysis was performed, and finally a mediation model was developed, including the path coefficients of the effects included in the mediational model, controlling for demographics, i.e., age, gender and socio-economic status.

## 3. Results

The sample was composed of 7649 participants (53.9% girls), aged between 10 and 23 years old (*M* = 15.05, *SD* = 2.36). Descriptive statistics for all study variables, as well as gender comparisons, are presented in Table 2.

Overall, participants reported relatively high levels of health literacy and percived social support, alongside moderate levels of stress and lack of well-being. Among the variables, relationships with teachers showed the lowest mean scores, indicating greater perceived difficulties.

Gender comparisons revealed statistically significant differences for all variables except health literacy. Compared to boys, girls reported higher levels of stress, greater difficulties in relationships with teachers, higher lack of well-being, and higher peer support. In contrast, boys reported higher levels of family support (Table 3).

Correlations among the study variables are presented in Table 4. Most correlations were statistically significant. Moderate positive associations were observed between stress and lack of well-being (*r* = 0.58, *p < 0.001*) and between difficulties in relationships with teachers and lack of well-being (*r* = 0.41, *p < 0.001*). In contrast, moderate negative associations were found between family support and lack of well-being (*r* = 0.42, *p < 0.001*) and between stress and family support (*r* = 0.41, *p < 0.001*). Regarding health literacy, the strongest associations were observed with stress (*r* = −0.31 *p < 0.001*) and family support (*r* = 0.30, *p < 0.001*).

### 3.1. Mediational Analysis

**Figure 1:**
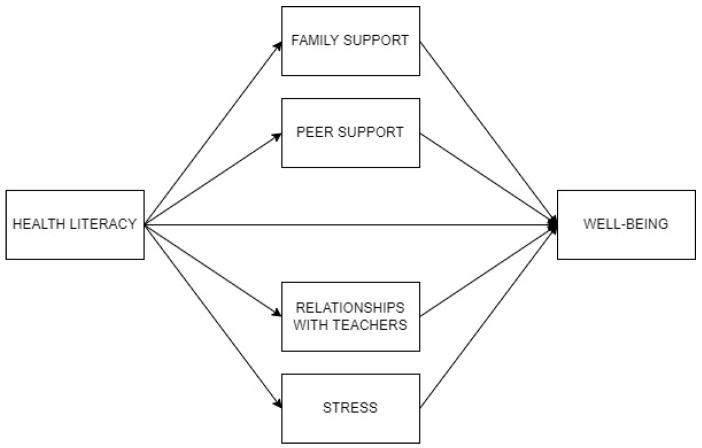
Partial mediation of family support, peer support, relationships with teachers, and stress, in the relationship between health literacy and well-being.

Figure 1 represents the mediation of family support, peer support, relationships with teachers and stress, in the relationship between health literacy and well-being. Regarding the results of the mediational analysis, presenting total, direct and indirect effects, as well as residual covariances, the results indicated a moderate negative total effect of health literacy on lack of well-being. After including the mediators, the direct effect of health literacy on lack of well-being was negative. The four mediators presented significant indirect effects, which explained the difference between total and direct effects. Thus, the effect of health literacy on lack of well-being can be partially explained by family and peer support, relationships with teachers, and stress. Health literacy influenced lack of well-being directly and through its negative indirect effects by these mediators. The greatest indirect effect was observed through stress. Residual covariances indicated the significant bidirectional associations among the mediators, in the same line as previous correlation analyses.

Concerning the path coefficients of the effects included in the mediational model, controlling for demographics, i.e., age, gender and socio-economic status, the results indicated that health literacy and family and peer support had negative effects on lack of well-being, while difficulty in relationships with teachers and stress had positive effects. Stress had the strongest effect on lack of well-being, while peer support showed the lowest effect. Moreover, health literacy had positive effects on family and peer support, and negative effects on difficulty in relationships with teachers and stress. The strongest effect of health literacy was observed on stress. Thus, the model indicated that more health literacy is related to more well-being directly and through its positive effects on family and peer support, and its negative effects on difficulty in relationships with teachers and stress.

Furthermore, some effects were found by gender, age and SES. Age differences were found in all study variables, meaning that older participants showed less health literacy and family support, but higher scores in peer support, difficulty in relationship with teachers, stress and lack of well-being. Gender differences were observed in all variables, except in health literacy. More family support was found in boys, while girls reported more peer support, difficulty in relationships with teachers, stress, and lack of well-being. Concerning SES, some differences were detected in health literacy, family and peer support, and stress. More SES is related to more literacy and both family and peer support, and less stress. Overall, the model reached an explained variance for well-being of 46.6%.

### 3.2. Moderations by Demographics

Table 5 presents the results of moderation by gender, age and socioecomic status (SES) on the associations between health literacy (L), family support (F), peer support (P), relationships with teachers (T), stress (S) and well-being (WB), with values representing standardized interaction coefficients (β). Gender was coded, with boys as 0, and girls as 1. Only statistically significant interaction effects (*p* < 0.05) are reported.

Concerning gender moderation, results indicated (a) a stronger effect of health literacy on family support and stress among girls; and (b) stronger effects of health literacy, family support, peer support, relationships with teachers and stress on well-being among girls. Regarding age, results indicated (a) stronger effects of health literacy, relationships with teachers and stress on well-being among younger participants; and (b) stronger effects of health literacy on peer support, relationships with teachers and stress among younger participants. No moderation effects were observed for SES in any of the examined relationships.

Health literacy showed a significant negative direct effect on well-being (β = −0.09, *p* < 0.001), while significant indirect effects were observed via family support (β = −0.03, *p* < 0.001), peer support (β = −0.02, *p* < 0.001), relationships with teachers (β = −0.04, *p* < 0.001), and stress (β = −0.11, *p* < 0.001). Stress presented the strongest association with well-being (β = 0.38, *p* < 0.001), followed by relationships with teachers (β = 0.16, *p* < 0.001), whereas family (β = −0.12, *p* < 0.001) and peer support (β = −0.08, *p* < 0.001) showed weaker effects. The total effect of health literacy on well-being was significant (β = −0.29, *p* < 0.001), with a significant total indirect effect (β = −0.20, *p* < 0.001). Moderation analyses indicated significant gender and age effects in several paths, whereas no significant moderation by socio-economic status was observed. The model explained 46.6% of the variance in well-being.

## 4. Discussion

There is a relationship between health literacy and well-being. Health literacy, associated with knowledge, skills and access to health, the adoption of health behaviors and the avoidance or better management of risk behaviors [4], is associated with a better perception of general well-being, associated with the perception of tranquility, feeling active, being emotionally excited and doing things with interest [30].

In this article, we analyzed the mediating role of personal factors (stress) and social support factors relevant to adolescents (relationships with family, friends and teachers) in this relationship between health literacy and well-being. As a result, we found that the effect of health literacy on well-being can be partially explained by support from family, peers, relationships with teachers and stress. A meta ethnographic study [10] also found a relationship between HL and social support.

We can therefore hypothesize that social support from family, friends and teachers, as well as stress management skills, will mean that health literacy can have an even stronger relationship with adolescents’ well-being. According to Michie and colleagues’ [31] model, for behavior change to occur, three factors must coexist: skills, motivation and opportunity.

Health literacy is closely linked to this model. Social support from people who are significant to the adolescent, together with their self-regulation skills (in relation to stressful situations), enhance and optimize knowledge, competence and motivation, and create opportunities to adopt healthier behaviors, prevent and mitigate risk and lead to a better perception of well-being, as corroborated by studies [2,3,4,5,6].

Taken together, the proposed model represents a conditional effects framework, in which health literacy is positively associated with well-being, and this association is moderated by social and personal resources. Specifically, the model hypothesizes that this effect is strengthened in the presence of high-quality relationships with family, friends, and teachers, and the effect is also strengthened among individuals with higher stress management skills. Analytically, this implies the examination of interaction effects between health literacy and each moderator in predicting well-being. The model aligns with an ecological and interactionist perspective, suggesting that health literacy alone may be necessary but not sufficient to optimize well-being, and that its impact depends on supportive social contexts and adequate personal coping resources [22,31,32].

Gender, age and socio-economic status differences are identified. Girls show greater stress, lower well-being and less support from family and teachers. However, they report a greater perception of support from friends when compared to boys.

In the case of age and socio-economic status, we see that older students and students with lower socio-economic status show fewer positive indicators when compared to younger students and those with higher socio-economic status, namely a lower perception of support from family, friends and teachers, as well as more stress, lower well-being and lower health literacy. The only exception is that there were no statistically significant differences in age in relation to the perception of support from friends.

The results are confirmed by previous research [7,8,22,23,24,33], but the integration of psychological, relational and sociodemographic variables into a single model to explain adolescents’ health literacy and well-being is a contribution of this study.

Regarding the moderating role, girls show a stronger effect of health literacy on family support and stress, and a stronger effect of all variables on well-being and a stronger effect of all variables on well-being. A study carried out [32] on social support found that it is positively predicted mental health literacy, positive coping style and mental health literacy. In addition, the relationship between social support and positive coping styles was moderated by social comparisons.

Younger students show a stronger effect of health literacy, relationships with teachers and stress on well-being, and a stronger effect of health literacy on relationships with friends with teachers and stress. No moderation was observed by SES in any relationship. Another study [34] concludes that gender (in favor of males) has a significant moderating effect on the relationship between health literacy, mental health, and attitude toward social support seeking among students. Age and educational level influenced health literacy and students’ mental health [35].

The study includes some limitations associated with the instrument used: the Health Behaviour in School-aged Children protocol is a general health screening scale for children and adolescents and does not use more specific measurement scales, particularly at the level of family, peer group and teacher social support variables. It is also a self-report scale, which can always lead to some bias, particularly in terms of the perception of SES. Additionally, cross-sectional studies have advantages (e.g., low cost, no significant ethical concerns, data collection at a single point), but they also have significant limitations [36]. In particular, cross-sectional studies do not allow for incidence estimates, there are difficulties with regard causal inference, and they prevent the analysis of temporal associations between outcomes and risk factors, which can make the interpretation of associations more complex [36].

From a socioecological perspective, our findings can be interpreted within a broader theoretical framework rather than as a simple restatement of results. Bronfenbrenner’s ecological systems theory emphasizes that adolescents’ development is shaped by multiple social contexts (microsystems such as family, peers, and school) interacting with the individual. Consistent with this, the mediating roles of family support, peer support, teacher relationships, and stress suggest that health literacy’s impact on well-being is realized through an interplay of personal and environmental factors. In other words, adolescents with higher health literacy may be better equipped to leverage supportive relationships and cope with stress, which in turn enhances their well-being, an interpretation that aligns with the idea that positive transactions between youth and their social environments foster healthy development [14,31]. Furthermore, the absence of any moderating effect of socio-economic status (SES) indicates that these processes hold similarly across different SES groups. The pathway from health literacy to well-being via social support and stress appears robust for adolescents regardless of their socio-economic background, implying that the benefits of health literacy and strong support networks are universal assets for youth well-being. This is theoretically significant because it suggests that strengthening individual capacities (like health knowledge and self-regulation skills) and bolstering adolescents’ support systems can promote well-being across the socio-economic spectrum, rather than only in advantaged groups. Practically, this lack of SES moderation means that interventions to improve health literacy and social support could yield positive outcomes in all socio-economic strata, potentially helping to reduce disparities in adolescent health outcomes [28]. In sum, by incorporating a socioecological framework into our interpretation, we provide a more critical and nuanced understanding of the findings—one that underscores how individual skills and surrounding social resources collectively shape adolescent well-being, and why the observed mediation and (lack of) moderation effects matter in both theoretical and practical terms.

## 5. Conclusions

The study concludes that health literacy is associated with adolescents’ well-being, and that well-being and health literacy should be understood from an ecological perspective [10,14], since they are related to different systems, such as individual adolescent factors, such as gender, age and stress management skills; and interpersonal factors, such as relationships with family, peers and teachers, which interact with each other. Finally, the socio-economic situation of adolescents is also relevant to their health literacy and perception of well-being [8,21,29].

In order to promote adolescents’ well-being, it is essential to have a multi-level intervention, developing universal awareness-raising and psychoeducation in relation to health behaviors, risk behaviors and strategies for developing socio-emotional skills. This universal approach should also involve the adolescents’ relevant contexts, such as school and family [37]. At a selective level, we identify groups most at risk in terms of well-being and health literacy, such as girls, older students and students with lower socio-economic status, who should have an intervention tailored to their specific needs. Finally, it is important to give opportunities to students, teachers and families who, because they show very low levels of well-being and health literacy, need a more individual and robust intervention [7,29].

The intervention should be planned and carried out from a multidisciplinary and multidimensional perspective, involving different ministries, organizations and professionals, such as health, education at its different levels from pre-school to higher education, social work, labor and justice.

## Figures and Tables

**Table 1:** Measures and variables under study measurements are part of the HBSC study protocol [26,27].

Variables	Measure
Gender	1—Male; 2—Female
Socio-economic status (SES)	FAS Scale: Family Affluence Scale, with 6 items that reflected the family’s material resources, such as owning a car or individual computer. The FAS score [28,29] was calculated for each adolescent based on the responses to these 6 items, on a scale ranging from 0 to 13 points, with the highest values indicating better financial level.
Well-being (WHO-5)	Scale with 5 items with scores from 0 to 5. A minimum score of 0 and a maximum score of 25. Higher values reveal a worst perception of well-being. The scale reveals good psychometric qualities, with a Cronbach’s alpha of 0.85 [25].
Stress	Scale with 4 items with scores from 0 to 5. A minimum score of 5 and a maximum score of 20. Higher values reveal greater stress. The scale reveals acceptable psychometric qualities, with a Cronbach’s alpha of 0.70 [25].
Family support	Scale with 4 items, on a 7-point Likert scale, with 1 being very strongly disagree and 7 very strongly agree. Higher values reveal greater family support. The scale has great psychometric qualities, with a Cronbach’s alpha of 0.95 [25].
Relationship with teachers	Scale with three items in five-point Likert scale, with 1 being totally agree and 5 being totally disagree. Higher values reveal a worse relationship with teachers. The scale has good psychometric qualities, with a Cronbach’s alpha of 0.84 [25].
Relationship with peers	Scale with three items, in five-point Likert scale, with 1 being totally agree and 5 being totally disagree. Higher values reveal a worse relationship with peers. The scale has good psychometric qualities, with a Cronbach’s alpha of 0.79 [25].
Health literacy	Scale with 10 items with scores from 0 to 5. A minimum score of 5 and a maximum score of 50. Higher values reveal a better perception of health literacy. The scale has good psychometric qualities, with a Cronbach’s alpha of 0.89 [25].

**Table 2:** Descriptive statistics of the variables (N = 7649).

Variables	Min	Max	*M* ± *SD*
**Stress**	4.00	20.00	11.04 (3.06)
**Family Support**	4.00	28.00	22.67 (6.63)
**Peer Support**	4.00	28.00	21.83 (6.37)
**Difficulty in relationships with teachers**	3.00	15.00	6.74 (2.54)
**Lack of Well-being**	5.00	30.00	15.07 (5.47)
**Health Literacy**	10.00	40.00	31.47 (5.08)
**Socio-economic Status**	7.00	19.00	13.87 (2.19)

**Table 3:** Comparison analysis according to sex.

	*Descriptive Statistics*				*Significance Tests and Effect Size*
	$\bar{x}$	*SD*	$\bar{x}$	*SD*	
	*Boys*		*Girls*		
*Stress*	*10.17*	*2.85*	*11.69*	*3.02*	*t (7288.980) = −22.108, p < 0.001, d = 2.94*
*Family Support*	*23.68*	*6.03*	*22.08*	*6.83*	*t (7346.248) = 10.675, p < 0.001, d = 6.47*
*Peer Support*	*21.69*	*6.33*	*22.16*	*6.26*	*t (7353) = −3.197, p < 0.001, d = 6.29*
*Difficulty in relationships with teachers*	*6.36*	*2.51*	*6.98*	*2.46*	*t (7353) = −10.642, p < 0.001, d = 2.48*
*Lack of Well-being*	*13.28*	*4.97*	*16.42*	*5.39*	*t (7313.600) = −25.977, p < 0.001, d = 5.20*
*Health Literacy*	*31.55*	*5.25*	*31.54*	*4.80*	*t (6940.002) = 0.106, p = 0.916, d = 5.01*

**Table 4:** Correlations.

	1	2	3	4	5	6	7	8
1-Age		−0.135 ***	0.116 ***	−0.162 ***	0.004	0.177 ***	0.247 ***	−0.047 ***
2-FAS			−0.114 ***	0.126 ***	0.065 ***	−0.032 *	−0.114 ***	0.113 ***
3-Stress				−0.407 ***	−0.242 ***	0.325 ***	0.582 ***	−0.307 ***
4-Family Support					0.373 ***	−0.320 ***	−0.424 ***	0.303 ***
5-Peer Support						−0.243 ***	−0.273 ***	0.269 ***
6-Difficult relationships with teachers							0.406 ***	−0.238 ***
7-Lack of Well-being								−0.305 ***
8-Health Literacy								

Note: *** p ˂ 0.001; * p ˂ 0.05.

**Table 5:** Moderation by demographics in the relationships included in the model.

	*β*	*t*	*p*	*LLCI*	*ULCI*
*Gender* *moderation on*					
*L- > WB*	*−0.06*	*−2.58*	*0.010*	*−0.10*	*−0.01*
*L- > F*	*0.09*	*3.99*	*<0.001*	*0.04*	*0.13*
*L- > P*	*−0.02*	*−0.92*	*0.359*	*−0.06*	*0.02*
*L- > T*	*−0.04*	*−1.62*	*0.104*	*−0.08*	*0.01*
*L- > S*	*−0.06*	*−2.69*	*0.007*	*−0.10*	*−0.02*
*F- > WB*	*−0.12*	*−5.43*	*<0.001*	*−0.16*	*−0.07*
*P- > WB*	*−0.07*	*−3.26*	*0.001*	*−0.11*	*−0.03*
*T- > WB*	*0.11*	*5.09*	*<0.001*	*0.06*	*0.15*
*S- > WB*	*0.15*	*7.55*	*<0.001*	*0.11*	*0.19*
*Age* *moderation on*					
*L- > WB*	*0.03*	*5.95*	*<0.001*	*0.02*	*0.03*
*L- > F*	*0.01*	*1.06*	*0.288*	*−0.01*	*0.01*
*L- > P*	*−0.02*	*−3.51*	*<0.001*	*−0.03*	*−0.01*
*L- > T*	*0.02*	*4.00*	*<0.001*	*0.01*	*0.03*
*L- > S*	*0.02*	*3.80*	*<0.001*	*0.01*	*0.03*
*F- > WB*	*0.01*	*0.94*	*0.348*	*−0.01*	*0.01*
*P- > WB*	*0.01*	*0.97*	*0.331*	*−0.01*	*0.01*
*T- > WB*	*−0.01*	*−3.26*	*0.001*	*−0.02*	*−0.01*
*S- > WB*	*0.01*	*3.60*	*<0.001*	*0.01*	*0.02*
*SES* *moderation on*					
*L- > WB*	*0.01*	*0.50*	*0.618*	*−0.04*	*0.06*
*L- > F*	*−0.03*	*−1.30*	*0.194*	*−0.08*	*0.02*
*L- > P*	*−0.04*	*−1.57*	*0.115*	*−0.09*	*0.01*
*L- > T*	*0.02*	*0.86*	*0.392*	*−0.03*	*0.07*
*L- > S*	*−0.02*	*−0.78*	*0.433*	*−0.07*	*0.03*
*F- > WB*	*−0.01*	*−0.41*	*0.685*	*−0.05*	*0.04*
*P- > WB*	*−0.03*	*−1.19*	*0.233*	*−0.08*	*0.02*
*T- > WB*	*−0.02*	*−1.03*	*0.305*	*−0.07*	*0.02*
*S- > WB*	*−0.04*	*−1.73*	*0.084*	*−0.08*	*0.01*

Note: Health literacy (L); family support (F); peer support (P); relationships with teachers (T); stress (S); well-being (WB).

## Data Availability

Data is not available for the HBSC consortium agreement.
